# (How) does RBF strengthen strategic purchasing of health care? Comparing the experience of Uganda, Zimbabwe and the Democratic Republic of the Congo

**DOI:** 10.1186/s41256-019-0094-2

**Published:** 2019-01-31

**Authors:** Sophie Witter, Maria Paola Bertone, Justine Namakula, Pamela Chandiwana, Yotamu Chirwa, Aloysius Ssennyonjo, Freddie Ssengooba

**Affiliations:** 1 Queen Margaret University, Edinburgh, UK; 2 School of Public Health, Makerere University, Kampala, Uganda; 3 Biomedical Research and training Institute, Harare, Zimbabwe

**Keywords:** Results-based financing, Performance-based financing, Strategic purchasing, Mother and child health care, Uganda, DRC, Zimbabwe

## Abstract

**Background:**

Results-Based Financing (RBF) has proliferated in health sectors of low and middle income countries, especially fragile and conflict-affected ones, and has been presented as a way of reforming and strengthening strategic purchasing. However, few studies have empirically examined how RBF impacts on health care purchasing in these settings. This article examines the effects of several RBF programmes on health care purchasing functions in three fragile and post-conflict settings: Uganda, Zimbabwe and the Democratic Republic of Congo (DRC) over the past decade.

**Methods:**

The article is based on a documentary review, including 110 documents from 2004 to 2018, and 98 key informant (KI) interviews conducted with international, national and district level stakeholders in early 2018 in the selected districts of the three countries. Interviews and analysis followed an adapted framework for strategic purchasing, which was also used to compare across the case studies.

**Results:**

Across the cases, at the government level, we find little change to the accountability of purchasers, but RBF does mobilise additional resources to support entitlements. In relation to the population, RBF appears to bring in improvements in specifying and informing about entitlements for some services. However, the engagement and consultation with the population on their needs was found to be limited. In relation to providers, RBF did not impact in any major way on provider accreditation and selection, or on treatment guidelines. However, it did introduce a more contractual relationship for some providers and bring about (at least partial) improvements in provider payment systems, data quality, increased financial autonomy for primary providers and enforcing equitable strategies. More generally, RBF has been a source of much-needed revenue at primary care level in under-funded health systems. The context – particularly the degree of stability and authority of government–, the design of the RBF programme and the potential for effective integration of RBF in existing systems and its stage of development were key factors behind differences observed.

**Conclusions:**

Our evidence suggests that expectations of RBF as an instrument of systemic reform should be nuanced, while focusing instead on expanding the key areas of potential gain and ensuring better integration and institutionalisation, towards which two of the three case study countries are working.

## Background

Over the last decade, results based financing (RBF) has been increasingly implemented in low and middle income countries, and especially in fragile and conflict-affected states (FCAS) [1]. Under RBF programmes, funds are made conditional on agreed outputs or outcomes, often with quality adjustments [2]. While research and evidence on RBF has grown since the first systematic review [3], it has mostly focused on the effectiveness of RBF and there remain some very significant gaps in our understanding of it, in particular in relation to RBF as a health system strengthening intervention [4]. In particular, supply-side RBF – sometimes called performance based financing (PBF), although here we use the term RBF - has been presented as a health system intervention with the potential to drive a more strategic approach to purchasing [5, 6]. However there has been limited empirical study of RBF’s actual impact on strategic purchasing arrangements in practice in low and middle income (and especially fragile) countries and settings. This article aims to start filling that gap by examining the experiences of three case study countries – Uganda, Zimbabwe and the Democratic Republic of the Congo (DRC) – all of which have adopted RBF in different ways and at different scales over the past decade. Improving strategic purchasing is increasingly seen as an essential step, and one of the most effective strategies to accelerate and sustain progress towards universal health coverage, as a way to get more value from the money invested in the health sector through improved health systems efficiency [7]. How strategic purchasing is understood and conceptualised varies in the literature, but we adapt a recently published framework, which outlines core functions that strategic purchasing implies by level – in relation to government roles, populations served, and providers [8]. We used this framework to inform our understanding of ‘strategic purchasing’ as well as to guide the data collection and analysis.

The three countries have experienced different types of fragility, conflict and crisis. The DRC has experienced periodic violence stretching back to colonial days, and more recently influenced by armed groups fleeing the Rwandan genocide, leading to humanitarian catastrophe in the east of the country, political upheaval and then stalemate [9]. Fragility features include the quasi absence of state services, such as justice, health care and security, for which local populations have to rely on a network of state and non-state actors [10, 11]. This has provided a policy vacuum in which non-government organisation (NGO) and donor-led experiments such as RBF could thrive. By contrast, Zimbabwe has had a single government since independence in 1980, but experienced prolonged economic and political crisis, culminating in hyper-inflation and collapse of public services in 2008. Resource constraints were amongst the main triggers for adoption of RBF in the health sector in 2011 [12]. Uganda had a general civil war ending in 1986 [13]. However, conflict continued in the northern region until the Lord’s Resistance Army, an insurgent group, was expelled in 2006 and peace talks began [14]. In this context, RBF was adopted to improve health services not just in areas recovering from conflict but across the country [15].

Table 1 provides an overview of the history of RBF schemes which are the focus of our study in the three settings.

**Table 1:** Summary of key features of RBF in the case studies

	DRC	Zimbabwe	Uganda
RBF adoption process	– RBF introduced since 2005 (earliest RBF adopted among the three cases) – First RBF project in South Kivu implemented by the NGO Cordaid – Followed by a number of other projects funded by the European Union, World Bank, USAID, and other NGOs in different provinces [1, 45].	– Since 2011 [12] – WB-funded and Cordaid-implemented pilot in two districts (Marondera and Zvishavane), then in an additional 16 districts – Scaled up to cover the entire country in 2014: HDF-funded and Crown Agents-implemented	– Since 2009 – Numerous RBF schemes, all donor funded, with the World Bank being one of the major donors [57–59], but also other schemes implemented by Cordaid in Jinja (2009–2015) and recently by the Belgian Development Agency, Enabel (formerly BTC) in West Nile and Rwenzori regions.
Main reasons of RBF adoption	Policy vacuum left room for NGO/donor-led experiments	Resource constraints as trigger for RBF adoption	RBF adopted to mitigate financial constraints in private sector and improve services across the country, including in the North
Focus of this study	EU-funded project (9th FED) and the ongoing World Bank-funded *Programme de Développement de Services de Santé* project (PDSS). The reason is that both schemes make use of newly-created semi-autonomous purchasing agencies (*établissements d’utilité publique*, EUPs – see Annex 1).	Both RBF schemes, covering the entire country	RBF pilots in the post-conflict northern region – World Bank’s Saving Mothers, Giving Lives (SMGL) (2012–2017) – DFID’s NuHealth (2011–2016) – USAID’s Strengthening Decentralisation for Sustainability (SDS) (2011–2017).
Impact evaluation	No impact evaluation published so far for the selected RBF programmes	An impact evaluation has been conducted by the World Bank in the original districts [42]. However, no independent research on RBF’s health system effects has yet been published.	Mid-term impact for SMGL shows a 30% reduction in maternal death. Other programmes are yet to be evaluated.

## Methods

### Study design

This study adopts a comparative case study design, and is qualitative and largely retrospective. In each of the settings, we focus on one or more RBF programmes, selected because of their relevance to our research question, in terms of strategic purchasing arrangements (for example, for the selection of RBF programmes in DRC) or in terms of relation to conflict-affected settings (e.g. for Uganda we have focused on programmes operating in the north). A set of common data collection and data analysis tools were developed at the beginning of the research, based on the analytical framework on strategic purchasing that we adopted (Table 2). Tools were then adapted at country level to better fit the context and type of data available. Data were collected through a series of key informant interviews and were integrated with analysis of documentation.

**Table 2:** Key actions for strategic purchasing in relation to different stakeholders within the health system

Key strategic purchasing actions by *government*	• Establish clear frameworks for purchaser(s) and providers
	• Ensure accountability of purchaser(s)
	• Ensure adequate resources mobilised
	• Fill service delivery infrastructure gaps
Key strategic purchasing actions in relation to *citizens/population served*	• Assess needs, preferences, values of the population to specify benefits
	• Inform the population of entitlements, establish mechanisms for complaints and feedback, publicly report on use of resources and performance
Key strategic purchasing actions in relation to *providers*	• Select (accredit) providers
	• Establish service agreements/contracts
	• Design, implement, modify provider payment methods to encourage efficiency and quality
	• Establish provider payment rates and pay providers regularly
	• Allocate resources equitably, implement other strategies to promote equitable access and monitor user payment policies
	• Develop, manage and use information systems, secure information on services provided, monitor/supervise provider performance and act on poor performance, audit provider claims, protect against fraud and corruption

Source: authors’ adaptation based on [8]

### Study sites

#### DRC

Data collection was carried out remotely, but refers to two RBF programmes carried out in the provinces of Kasai Occidental and Oriental, North Kivu and Province Orientale (EU-funded FED programme), and Katanga, Équateur, Bandundu and Maniema (World Bank-funded PDSS programme).

#### Zimbabwe

Data collection was done at national level and in two provinces (Midlands and Mashonaland East), including four districts (Murewa, Marondera, Gokwe North and Gokwe South). These provinces were selected as they were the sites for the frontrunner districts in 2011. The districts were chosen as representing one each from the two schemes (Cordaid and Crown Agents) per province and including the two original pilot districts [16].

#### Uganda

Primary data collection was conducted at the national and sub national levels. For the sub national level, districts in Acholi and Lango sub-region, where the NuHealth, SDS and SMGL schemes had been implemented, were selected. The study districts included Gulu, Amuru, Kitgum, Lira and Oyam.

### Data collection

#### Document review

Documents concerning the three countries of analysis were gathered based on previous studies and research carried out by the authors [10, 15, 17] as well as through direct knowledge of the context. Moreover, key informants were asked to provide additional documents as relevant. Documents reviewed included a few published articles, as well as unpublished documents relating to the health sector (e.g., policies and strategies) and RBF documents (e.g., implementation manuals, sample of contracts, list of indicators, internal and external evaluations, presentations, and annual reviews). In total, 23 documents were reviewed for DRC, dating between 2008 and 2017. For Zimbabwe, 60 documents were reviewed, dating from 2008 to 2018, the vast majority of which were operational and grey literature. In Uganda 27 documents were reviewed, dating between 2004 and 2018.

#### Key informant interviews

Key informants interviews were carried out in all settings using similar semi-structured interview guides. The guides were developed based on the elements of the framework that we adopted (Table 2) and were adapted in each setting to better capture the specificities of the context and of the interviewing process. In each setting, the interviewees were selected purposefully, aiming to be as comprehensive as possible of all the actors involved in the RBF programmes considered. However, there are some variations in the number of respondents across the three countries which are due to the availability and accessibility of key actors in the different settings.

##### DRC

For the DRC, 9 key informant interviews were carried out in March and April 2018, with representatives of the MoH at central and decentralised level (*n* = 2), EUP staff (*n* = 2) and international technical assistants involved in the support to and implementation of RBF programmes and EUPs in particular (*n* = 5). The selection aimed to cover as much as possible those involved in the RBF programmes considered, and specifically in the establishment and management of the EUPs as purchasing agencies. Some interviewees were identified based on their roles, while others were contacted based on suggestions from previous key informants. Interviews were carried out in French and remotely, via phone, WhatsApp or Skype. Interviews were recorded and detailed notes taken.

##### Zimbabwe

Forty key informant interviews were conducted for Zimbabwe between February and March 2018. They included Ministry of Health staff at national (*n* = 5), provincial (*n* = 6) and district levels (*n* = 7); staff from other public bodies and ministries (*n* = 3); development partners (*n* = 10); RBF consultants (*n* = 3); and RBF implementers (*n* = 6). Purposive sampling was used to identify key informants at national, provincial and district levels, based on their knowledge and involvement on RBF from its inception till the present. The selection of interviewees was as comprehensive as possible, including individuals currently holding RBF-related posts or who were previously in such positions. A number of relevant organizations, groups and individuals involved in RBF were preliminarily identified. New individuals were added based on the results of the documentary review or as suggested by key informants.

Key informants were interviewed in English. Most interviews took place in the informant’s place of work, but in a location where privacy was assured. Some interviews were conducted by phone or Skype, where physical distance or access necessitated it. Interviews focused on the period from 2008 (prior to RBF introduction) to present and were tailored to the time available and the knowledge of the KI. Interviews lasted from 30 min to two hours, with an average of one hour. Interviews were recorded where informants were comfortable with that, and detailed notes were taken.

##### Uganda

A total of 49 interviews with key informants were analysed for the purpose of this study, including from the Ministry of Health (*n* = 4); Ministry of Finance/Presidency (*n* = 2); development partners (*n* = 6); non-governmental organisations and implementers (*n* = 13; consultants and auditors (*n* = 3); district local government staff (*n* = 6); and facility managers (*n* = 15). Thirty-five were composed of transcripts of earlier key informant interviews which were reanalysed. These interviews were conducted by Makerere University School of Public Health in 2015 [15]. An additional 14 interviews were undertaken in 2018 in order to focus on questions relating to the impacts on strategic purchasing, with a greater emphasis on the experience in northern Uganda. The interviews lasted from 35 min to two hours. All the 14 additional interviews were recorded, except two. In these cases, the participants were uncomfortable with being recorded and preferred that the research team takes only notes.

### Data analysis

Data analysis was done iteratively. A first analysis of the documents collected was conducted before the interviews in the field, and guided the discussion during interviews. Later on, new documents were added to the review, and a final round of analysis carried out. For all countries, both documents and key informant interviews were analysed using thematic analysis. The initial analysis was carried out separately by each country team and consisted in coding the text (documents or interview notes or transcripts) based on the predefined list of categories which identified the key elements of strategic purchasing in relation to government, populations and providers in our analytic framework [8], which was modified to reduce the categories of analysis and adapted to the specific contexts (Table 2). Data analysis was carried out manually for Zimbabwe and DRC and using Atlas ti version 7.0 for Uganda.

During a workshop in June 2018, the teams shared their findings and prepared a three-country matrix, comparing the key results against each of the elements of strategic purchasing identified in the table. The comparative matrix and the team discussion that followed enabled comparison across cases (reported in the findings section), identifying common patterns and differences and deriving higher level conclusions, which are presented in the ‘discussion’ section.

### Ethical approval

Ethics approval was obtained from Queen Margaret University’s Research Ethics Panel. Additional ethics clearance for primary data collected at country level was also granted by the Makerere University School of Public Health, Ethics Review Board, and the Uganda National Council for Science and technology (SS4500), as well as the Medical Research Council of Zimbabwe (MRCZ/A/2265). The study also received authorisation from the MoHCC in Zimbabwe.

## Results

The findings section first provides some background on the RBF programmes included in this study, focusing specifically on the strategic purchasing arrangements it established or modified. Secondly, it presents the results of our analysis in relation to the key actions for strategic purchasing, following the structure of Table 2 above.

### Overview of RBF programme and its role in purchasing

#### DRC

In the DRC, we specifically looked at two of the RBF programmes that have been implemented recently. The 9th FED programme was funded by the European Union from 2005 to 2010 (another EU-funded project followed but under different design and arrangements, not explicitly considered here). It was Implemented in the provinces of Kasai Occidental (where it covered 16 Health Zones), Kasai Oriental (21 zones), North Kivu (15 zones) and Province Orientale (12 zones).The *Programme de Développement de Services de Santé* (PDSS) is mainly funded by the World Bank with contributions also from Global Fund, UNICEF, UNFPA, USAID, GAVI. It started in 2017 in the provinces of Katanga, Équateur, Bandundu and Maniema,^1^ with the aim of covering 140 zones in total. Broadly, the aim of both RBF programmes was to inject funding in an extremely underfunded and cash-strapped system to improve the quality and also the accessibility and coverage of health services.

The design of the two RBF programmes is slightly different, but both make use of contracts with public and faith-based facilities and health authorities at zonal (Zonal Health Teams - *Equipes Cadre de Zone,* ECZ) and provincial level (Provincial Health Divisions - *Divisions Provinciales de Santé*, DPS) to provide health or health management services as defined in the contracts in exchange of a cash payment made to facilities which can be used to cover staff bonuses and facility’s running costs and small investments [18]. However, both programmes also included a component of non-performance based support in cash or in kind [19].

The specificity in the design of both RBF programmes compared to others in the DRC and elsewhere is the creation and use of a semi-autonomous purchasing agency, established at provincial level. These agencies are commonly called EUPs based on the acronym of ‘*établissements d’utilité publique*’ (EUPs, public service agencies – Annex 1).^2^ Their creation as an innovative institutional model was “a bit of an accidental decision”, according to key informants. Initially, the EUPs were created by AEDES, the international implementing agency of the FED project, in order to respond to the need to reconcile EU procedures and fiduciary concerns with the preference for national structures, ownership, and long-term sustainability. Based on this, the EUPs were created by AEDES in 2008 as semi-autonomous entities, delegated by the Ministry of Health and the Ministry of Finance to implement a public mission, namely to manage EU funding to purchase health services. The key informants involved in the creation and early management of the EUPs stressed how the EUPs were envisaged to move fund pooling and management to a decentralized level, with the double aim of improving flexibility and autonomy at that level in the DRC, where the central level struggles to control the periphery [9], but also to strengthen accountability and trust in the financial system. As a semi-public body with representatives of government, donors and civil society, the EUPs were seen as having this potential. Many viewed the EUPs as a potential tool for pooling and channelling funds to health facilities from different sources, including multiple donors (a sort of provincial basket fund), but also from government at national and provincial levels, as well as from health insurance or *mutuelles*, effectively becoming the sole purchasing agency in the provinces.

#### Zimbabwe

While the experience in DRC is one of donor projects establishing new structures at provincial and district levels, in Zimbabwe, RBF developed in the context of a national system which, though battered by economic and political crisis, retained its integrated system, which had been one of the stronger performers in Africa pre-crisis [16]. The initial model was developed by the Ministry of Health and Child Care (MoHCC) in partnership with the World Bank and Cordaid, and guided by a national RBF steering committee. The RBF programme funded 16 reproductive, maternal and child health (MCH) indicators at rural health centre (RHC) level and five at referral level, with payments linked to number of outputs, with additional payments for quality and remoteness [20]. It scaled up relatively quickly from the initial 18 districts to the whole country in 2014. The expansion was implemented by Crown Agents, funded from a donor pooled fund, managed by UNICEF. It is focused on rural areas, covering all 60 rural districts and two urban districts [20], with the cities of Harare and Bulawayo excluded. Until 2018, district hospitals were also excluded from the RBF programme in the 42 Crown Agent districts, though contracts have been signed in 2018 for certain MCH referral indicators to be paid at district hospital level in all RBF areas.

The aim was for RBF to operate within existing national structures, however, due to lack of trust between development partners and government, and arrears which were outstanding from the Government of Zimbabwe to the international institutions, funding of RBF had to be channelled through international organisations [12], which provided the fund-holding, contracting, verification and technical support roles. In relation to verification, in the Cordaid programme, the local field officer originally provided the first-line verification, followed by external checks by the University of Zimbabwe until late 2017. The community sisters (based at the district) are now responsible for monthly verification in both schemes, though many report logistical challenges to conduct these as regularly as expected [16]. Counter-verification is conducted by the implementing agency Health Field Officers, who also conduct quarterly exit interviews to assess community satisfaction (in the Crown Agent districts). Community-based organisations are contracted to undertake these in Cordaid districts, to maintain more separation of functions.

District Health Executives undertake quarterly quality checks, using an integrated checklist, and RBF has provided resources to support supervision at provincial and district levels. The Provincial Health Executives are paid against four indicators, which focus on administrative tasks in relation to the RBF programme. District Health Executives have 12, which are a mix of performance-related and administrative [20]. RBF is also embedded in wider national institutions. The district-level RBF steering committees are meant to meet quarterly and to report to the District Development Committees [20], but vary in their level of engagement.

In 2017–18 a process of institutionalisation began, whereby these purchasing functions started to be transferred to a semi-independent project implementation unit (PIU) in the MoHCC for the 18 World Bank/Cordaid districts. During the initial period, staff will be transferred from Cordaid, to retain their expertise, and posts will be externally financed. Meanwhile, the Ministry of Finance commitment to RBF has increased, although the financial sustainability of the programme remains uncertain [12].

#### Uganda

In Uganda, the RBF schemes that were assessed Included Saving Mothers, Giving Lives (SMGL) (2012–2017), NuHealth (2011–2016), and Strengthening Decentralisation for Sustainability (SDS) (2011–2017). These schemes were set up with funding from the World Bank, DFID and USAID respectively and covered selected districts, with a focus on Western and Northern Uganda. Project implementation units acted as fund holders, which made payments for outputs to private not-for-profit (PNFP) facilities. The performance verification functions were outsourced to separate entities – mostly international companies such as Price Waterhouse Coopers, Health Partners International and Montrose. The verification agencies worked with local NGOs and local governments in this function [15].

Most pilots have been implemented in the private sector, particularly PNFP/mission facilities, with the public sector facilities being considered only in the recent past. In terms of service packages, most schemes focused on maternal and child health care services, immunization and outpatient care services, a selective benefit package within the Uganda minimum health care package [21, 22]. A few schemes supported district local governments to undertake supervision, planning and management related tasks as the basis for payment of these units. Over time, more support is being provided to local government and central government to support integration of RBF at national and district level [23]. A national RBF framework has been developed to customise RBF functions to national and district levels in accordance with the decentralisation policy of health services in Uganda. Many functions that were previously undertaken by international and local NGOs have recently been integrated within MOH and District Health Management Teams [24]. Currently, RBF schemes are being scaled up using World Bank grants to 78 districts of Uganda over the next five years [23]. The current RBF schemes (i.e. World Bank roll out and BTC^3^) are working with both PNFP and public sectors, as opposed to the previous ones, which only focused on the PNFP sector [23, 25].

### Effects on key strategic purchasing actions

In the following sections, we review the effects of RBF on the key strategic purchasing actions identified in our analytic framework. A summary of the key findings is provided in Table 3 below.

**Table 3:** Summary of key findings

		DRC	Zimbabwe	Uganda
Key strategic purchasing actions *by government*	Establish clear frameworks for purchaser(s) and providers	- Weak regulatory capacity - RBF contracts provided clearer rules and regulations, though re. RBF funding only	- Strong regulatory frameworks (e.g., Results Based Management since 2005), but resource-starved. - Only primary level and some indicators covered	- RBF did not radically change regulatory frameworks - Some changes only for providers/services covered by RBF
	Ensure accountability of purchaser(s)	- EUPs have stronger accountability links with MoH compared to NGO projects - In practice, government/MOH did not exercise their oversight role	- Parallel system with external purchasers - Accountability of purchasers to funders as well as to government - Non-RBF funding through different channels	- RBF operating in parallel - Plans for a national scheme under MoH leadership is being used in the current RBF model.
	Ensure adequate resources mobilised	- Out of pocket payments main source of funding - RBF mobilised additional resources to decrease user fees - Limited success of EUPs in raising/pooling funds	- RBF provided modest but partially additional funds, still significant for primary care providers - Focus on MCH indicators - Donor dependent	- RBF donor funded, with donors working in silos even within the same region - Discussions of a virtual pool but not realised yet
	Fill service delivery infrastructure gaps	- Assessments carried out by RBF projects and bonus provided in some cases	- RBF provided some upfront investment, but no major revision of infrastructure planning in relation to needs	- District teams remain responsible for identifying service delivery infrastructure gaps
Key strategic purchasing actions *in relation to citizens/population served*	Assess needs, preferences, values of the population to specify benefits	- Norms on activity packages existed and RBF worked within them, covering some services in the packages - EUPs allowed to revise RBF package – but rarely done in practice	- No consultations on needs, values and preferences - Package defined nationally with no scope for variation at local level	- No direct consultation with communities - Needs determined using routine data and national surveys and indicators - RBF includes services from the minimum package
	Inform the population of entitlements Establish mechanisms for complaints and feedback Publicly report on use of resources and performance	- RBF requires price list to be made public on the facility wall - RBF aimed at improving community participation by strengthening Health Management Committees - Community verification, but delays in data collection and no/little analysis and feedback - IT portal to report performance, but only for RBF indicators and no community verification scores	- RBF requires price list to be made public on the facility wall - RBF helped revive Health Centre Committees: variable results and capacity	- Pre-existing mechanisms for feedback (barazas, suggestion boxes, Health Unit Management Committees) - Client satisfaction surveys in some RBF programmes
Key strategic purchasing actions *in relation to providers*	Select (accredit) providers	- Done by health authorities/ regulator, EUPs have limited power in deciding which facilities to contract (limited to type of contract or sub-contracts) and to enforce sanctions	- RBF did not change existing accreditation system - RBF required facilities to meet minimum criteria, including developing an operational plan, having a bank account and a functioning HCC	- Accreditation bodies preexisted and RBF did not change this. - A few schemes have provided start-up capital to enable more providers to get accreditation requirements.
	Establish service agreements/contracts	- RBF introduced contracts – but rarely enforceable with limited room for sanctions - Contracting done by EUPs, and limited to RBF services/facilities	- RBF introduced contracts – but rarely enforceable with limited room for sanctions - Contracts are limited to services and facilities covered by RBF	(As in Zimbabwe)
	Design, implement, modify provider payment methods to encourage efficiency and quality	- Very little public funding other than (some) salaries - RBF provided additional performance-based funding, but did not alter public/other donors’ funding - Some evidence of quality improvements	- Mixed picture in terms of outputs and quality improvements - Focus on MCH services, including some for which coverage is high - Some quality improvements (e.g., drugs availability)	- Little quality improvements given broader structural challenges such as workforce shortages and insufficient medicines distributed from the center. - Private facilities have more flexibility to improve service inputs.
	Establish provider payment rates Pay providers regularly	- RBF introduced payment rates for services (not the practice before) - Rates are additional to user fees - Rates defined at provincial level, depending on funds available and donors’ preferences (FED) - Rates defined centrally and included in Project Manual (PDSS) - Delays in paying providers	- RBF introduced payment rates for services (not the practice before) - Rates defined centrally, focus on MCH and low coverage indicators - Concerns over sustainability of payments (rates have been reduced over time)	- RBF introduced payment rates for services (not the practice before) - Payment methods complex and not well understood - Different schemes have different indicators and rates, depending on funders‘preferences and budget - Frequent unilateral decisions by fund-holders, often poorly communicated to provider and local governments
	Allocate resources equitably Strategies to promote equitable access Monitor user payment policies	- Bonus to compensate remote facilities - Extra funds to cover services provided to the very poor (*Equity Funds*), but only for hospital services (FED) and for few services (PDSS) - Support to reduce user fees and introduce *flat fees* to cross-subsidise between patients - Community verification to monitor user fee payments	- Remoteness bonus, but considered too small and failed to compensate facilities with small catchment areas - RBF aimed to remove user fees for the services it covered. However, no difference in out of pocket payments between control/intervention areas (in impact evaluation)	- No bonus in payment calculation but some initial bonus to remote facilities. - Facilities/districts often chosen as easier to work with, adding to the fragmentation and inequity - Reduction of user fees (in PNFP facilities) as a precondition for RBF support
	Develop, manage and use information systems to monitor/audit performance and protect against fraud Supervise providers	- RBF information system is parallel to HMIS. Plans to ensure integration in the future - Zonal/Provincial teams contracted to ensure supervision	- RBF used HMIS data after having verified and corrected it - Providers have multiple data reporting requirements - RBF brought greater focus on data quality - Little evidence of false claim, risk based verification - Pre-existing well developed and integrated supervision system to which RBF provided funding	- Similar issues of multiple data streams, but HMIS remains main one - Supervision system only partially affected/funded by RBF

### Effects on key strategic purchasing actions by government

#### Establish clear frameworks for purchasers and providers

In contrast with the other two settings, in DRC there is very weak regulatory capacity of the state at all levels (central, provincial and zonal). The introduction of RBF brought the establishment of contracts between purchasers and providers, which provided clearer rules and regulations for the providers. Additionally, contracts were also signed between different levels of the MoH hierarchy. However, these frameworks and regulations (as established in the contracts) refer to the RBF funding only and do not apply to other funds.

In Zimbabwe, the regulatory frameworks existed prior to RBF, but were resource-starved and operated within an integrated hierarchy – for example, Results-Based Management (RBM) was introduced in 2005 across the public sector but was never fully operationalized. Under RBM, performance contracts are established at each level, however, the resources to accompany the targets did not materialise [16]. With RBF, contracts were signed by the implementing agencies with provincial, district and RHC levels, establishing roles and payment systems, but the difference was the availability of funds to support the realisation of these contracts. As with DRC, the RBF purchasing body remained external, at least to date, and covered a sub-set of services, and was focused at the primary level.

In Uganda, as with Zimbabwe, there was an extensive planning and regulatory environment, which RBF has not changed radically. RBF worked within the existing system and focused on a sub-set of services and activities. Some RBF schemes set up their own parallel institutions (fund holder, auditors/verification agents, and implementing agencies). In the newer schemes, such as BTC one and World Bank roll out, there has been more explicit effort to work with the Ministry of Health and district health teams and leaders [23, 25].

#### Ensure accountability of purchasers

In DRC, accountability mechanisms are generally very weak or non-existent. The creation and use of EUPs as purchasing agencies appears to ensure accountability and strong links with the MoH (for example, compared to the use of an external NGO or implementing agency) since there is a clear ‘delegation of public functions’ from the MoH to the EUP. However, in practice, some key informants noted that the government did not exercise actively its oversight function under the FED programme, and this was left to the implementing agency (AEDES) and its technical assistants. This may improve under the PDSS since the national RBF unit embedded within the MoH is in charge of overseeing and supporting the EUPs. In general however, the EUPs remain parallel structures and their establishment does not affect the broader, pre-existing systems of accountability (e.g. accountability between levels of health authorities, or accountability of other purchasers, such as NGOs supporting services at local level, but not involved in RBF).

In Zimbabwe, RBF has also established parallel systems for purchasing RBF indicators, and the accountability of the purchasers is to funders as much as to the government. Moreover, the bulk of purchasing in the wider public sector remains largely unaffected by RBF. The large proportion of public resources continue to be spent on staffing and a number of the health system pillars have their own purchasing arrangements (e.g. for staff and for pharmaceuticals), rendering public purchasing underpowered and fragmented. However, this has not been altered by RBF. The bulk of aid funds are managed by a few organisations, such as UNICEF and UNDP. There is a national steering committee which coordinates between pooled fund donors (previously a larger list but now including DFID, the European Union, SIDA and Irish Aid) and the Ministry of Health, however, these pooled funds are now a small proportion (7%) of the overall aid funding to the health sector [17].

In Uganda, RBF has been operating in parallel to and has not yet impacted on the wider accountability of purchasers, though this may change if funding is brought back from NGOs into payments through a unit in the MoH, as planned under the World Bank programme. More generally, the sector-wide approach created structures between government, donors and civil society to improve accountability [26]. Furthermore, the Ministry of Finance has gradually implemented an Integrated Financial Management system in order to improve accountability within government ministries and local governments [27].

#### Ensure adequate resources to meet service entitlements

Patients are the main source of health financing in the DRC - 40% of total health expenditure, based on 2015 national health accounts, compared to 37% from donors and 17% from government - [28] - and are expected to pay for all the services they use, except a few preventative services vertically funded and in areas and times of acute crisis (for services provided or supported by NGOs). Usually, donors and NGOs decide which services to provide for free, where and to whom and mobilise funds for it. The MoH plays a little role for the coordination of resource mobilization at central level (for large donors) or at local level (for smaller donors and NGOs).

RBF has mobilized additional resources from international donors interested in funding service delivery through this mechanism (with a budget of $1.5 per capita for the FED programme [19] and slightly less than $3.70 for the PDSS, in its early phases – personal communication). The vision under both RBF programmes was that the EUPs would play a stronger role in mobilizing even further resources from other donors, but also from government, by providing a trusted semi-autonomous body which could meet the fiduciary concerns of donors. EUPs were envisaged to become a basket funding mechanism (to pool, but also to mobilise funding) at provincial level. With time, EUPs might also work as strategic purchaser and fund-holder for funds from *mutuelles* and social health insurance. However, in reality this has not happened in the way envisaged. Under the FED programme, only UNICEF decided to use the EUPs to pool and channel funds for their water and sanitation (WASH) projects, but this remained in parallel to health sector funding from the EU. Under the PDSS programme, there is more participation from a number of donors (World Bank, Global Fund, GAVI, USAID, UNICEF, UNFPA) but this is negotiated between donors in Kinshasa or internationally, rather than at EUP level. The plan for the integration of *mutuelles* has been discussed in North Kivu (one of the better-working EUPs), but never really took off. Discussions to include funds from the provincial government in North Kivu have not yet borne fruit.

For Zimbabwe, RBF provided modest but partially additional funds: the original RBF scheme was budgeted at $2 per capita [29] and a recent study estimated RBF’s incremental cost at $3.19 per capita [30]. This is small in the overall scale of expenditure – public health expenditure, including aid but excluding out of pocket payments – is estimated at $69 per capita [17] – but still significant as a revenue source at primary facility level. As in DRC, there was an aspiration to mobilise other funders to pool revenues with RBF funds, However, that has not happened as yet. RBF remains focused on maternal health indicators in particular, and is not able to support other major population groups, such as chronic patients, those with communicable or non-communicable diseases, or important population health priorities, such as nutrition, environmental health or mental health [16]. RBF funds are subject to annual commitments by donors which have reduced over the years and become more insecure. Given the on-going economic and fiscal challenges in Zimbabwe, resource mobilisation remains inadequate; household payments accounted for around 25% of total health expenditures in 2015, of which 95% were out of pocket [31].

In Uganda, the Ministry of Health is responsible for the core functions of resource mobilisation as well as policymaking, standards formulation and quality assurance [32]. The major funding sources for health are out of pocket expenditure (50% of the total), off-budget/on-budget donor contributions (35%) and government (15%) [33]. The health sector has remained inadequately and irregularly funded [34]. Donors, who have been working in silos, even in the same region, have funded the majority of the RBF schemes - for example, DFID funded Nuhealth, while USAID funded the SDS scheme in Northern Uganda. Per capita expenditure for each scheme is not reported. As in Zimbabwe, there has been talk about creating a virtual pool through RBF, to bring donor funds together, however, there is no evidence of this happening to date.

#### Fill service delivery gaps

In DRC, national standards exist for infrastructure (e.g. facilities per inhabitant), staffing and equipment [35]. However, many of these standards remain theoretical because of lack of resources [36]. In order to address infrastructure gaps, both RBF programmes carried out initial assessments to make sure basic equipment, infrastructure (and possibly staff – but without being able to influence the hiring of health workers) was in place. The PDSS also provides cash payments (*unités d’investissement*) to facilities to cover their investment needs as detailed in the facilities’ business plans [18].

In Zimbabwe, RBF provided some upfront investment for primary care facilities and also supports on-going repairs and upgrading through facility reinvestment of funds. However, the programme has not led to any revision of infrastructure planning in relation to population needs [17], as it is a system in which funds flow according to utilisation, which largely reflects catchment populations [37], rather than following an assessment of how best (most equitably and efficiently) to meet population health needs.

In Uganda, as in Zimbabwe, district health teams are mainly responsible for supervision and identifying service delivery gaps. This system was unchanged by the RBF programmes, but some of the RBF projects (such as NuHealth, BTC, and SMGL) conducted facility assessments at their start and in most case provided seed grants to improve functionality before enrolment into the schemes.

### Key strategic purchasing actions in relation to population served

#### Assessing service needs, preferences and values of the population and using them to specify service entitlements

Across the three countries, RBF worked within defined national entitlements and did not involve new consultation around care packages or specification of needs. In DRC, there are national level norms for services to be covered within the ‘minimum package of activities’ (PMA) for health centres and in the ‘complementary package of activities’ (PCA) for hospitals. Aside from these, there are no national-level, specific standards of care or clinical guidelines in the country [38]. The RBF programmes have adapted to this and both schemes covered most of the services included in the PMA/PCA. The PDSS Operational Manual suggests that these could be modified “if the regulators (health authorities) consider other services as a local public health priority” [18]: p.51). However, this has not been done in practice and it is not clear who would propose and have a final decision on this – whether it would be the EUPs (as strategic purchaser), the Provincial Division of Health (DPS) or the central level (Cellule Technique FBR), according to key informants.

Equally, in Zimbabwe, there has been no consultation linked to RBF on users’ needs or preferences to feed into the benefits package, as this is agreed nationally and there is no scope for variation at local level. Similarly, in Uganda, there was no structured process for consultation with communities in relation to selection of services, as the majority of the RBF schemes derived their service package from the minimum health care package [22].

#### Informing the population of their entitlements

Some efforts to strengthen community information and participation are typically made under the RBF programmes. For example, in DRC the PDSS requires that the price list for users is made public on the wall of the facility and known by the community [18]. This is also the case in Zimbabwe.

Additionally, in DRC RBF programmes aimed at improving community participation by strengthening the role of the *Comités de Gestion de Santé/Comité de Direction* (the latter is the hospital level one) and *Comité de Développement de la Santé*. For example, under the PDSS programme, these bodies are involved in the preparation of the business plans (or management plans) of the facility, they participate in the decision over the fees to be applied to each service and the organisation of regular meetings with the health committees is one of the score criteria in the quality check list. In Zimbabwe, RBF has helped to revive Health Centre Committees and has shifted their role from fund-raising to revenue allocation. However, studies highlight many challenges in relation to their role of connecting with and raising awareness amongst communities and also their variable capacity [39, 40].

Both RBF programmes in DRC introduced ‘community verification’, which is carried out by local associations. These associations are contracted by the EUPs, who are also in charge of organising the community verification (for example, by providing the sample of patients to be visited). Community verification is aimed at checking the real existence of patients indicated in the facilities’ registries, but also to assess their satisfaction, services received and fees paid. This information is fed back to the EUP but, again, echoing findings in Zimbabwe, there have been delays in the collection and analysis of data from the communities [19] and it is not clear how far the information is shared back with communities or what is done at facility level to respond to low scores or complaints. In summary, for both DRC and Zimbabwe, how effective design elements, such as community verification and strengthening of health committees, are in strengthening the link with the population and ensuring their awareness and access to entitlements is not clear, as also confirmed in other studies [10, 41].

Under the PDSS programme, there is a publicly available IT portal^4^ showing RBF results. However, it only focuses on RBF indicators and measures and does not report on community verification scores.

In Zimbabwe, one of the elements enforced by RBF is grievances boxes in health facilities, which might increase responsiveness, however, studies have found that there is low use of complaints mechanisms because of fear, low awareness, and tolerance of facility conditions [39, 42]. There is a feedback mechanism through client satisfaction surveys, which account for 20% of the quality scores in calculating RBF payments. However, results tend to be high across the board, suggesting a lack of sensitivity to quality [17], and again, it is not clear how they feed back into quality improvement.

In Uganda, various mechanisms for receiving feedback already existed prior to RBF. These included Health Unit Management Committees, suggestion boxes, ‘Barazas’ (community feedback dialogue meetings), and village health teams. A patient charter was developed to ensure that the rights of patients are protected in the course of seeking health care and that patients can demand their rights to quality health care [43]. These have not been optimally used due to inadequate funding.

In some RBF schemes (Cordaid and NuHealth) client satisfaction surveys were integrated into the design to capture service users’ perspectives, feeding into quality bonuses as in Zimbabwe. Health assemblies and regional meetings were also held to provide learning and information sharing opportunities among different stakeholders Other schemes in Northern Uganda utilised media such as local FM stations and meetings with various stakeholders to share information with the local community and beyond. It is not clear how often and to what extent the feedback is acted upon but in some cases key informants indicated that improvements were made.

There were already national guidelines on public reporting of expenditures by budget line at facility level in Uganda [44], which RBF also mandated (Cordaid, NUHealth, BTC). Health facilities are also required to publicise (pin on their noticeboard) their performance in relation to services such as immunisation. RBF did not change this, rather it added extra indicators that facilities needed to display. Some of the schemes were reported to have done random selections of mothers to conduct exit interviews with them during the supervision visits. Other facilities selected ‘ward leaders’ among patients to provide feedback on behalf of patients.

### Key strategic purchasing actions in relation to providers

#### Selecting or accrediting providers

In DRC, selection of providers is done by the regulator (the health authority) at the time when they prepare the *carte sanitaire* (mapping of all the health facilities in an area). At that stage, the health authorities decide which facility is the reference facility for the area based on the population size. RBF did not bring substantial change to this. Accreditation and selection are still done by the health authority based on national *Normes sanitaires* and the local *carte sanitaire*. EUPs have limited power in deciding which facilities to include in the contract, according to key informants, and therefore also to enforce the potential sanction of not contracting an under-performing or gaming facility. The only decision EUPs had under the FED project was in terms of deciding which type of contract to offer to facilities (*intégration* or *progression,*) based on their infrastructure and equipment levels. Initially, facilities with an integration contract would receive payment only for curative consultations provided and were paid with drugs (rather than cash) [45].

Under the PDSS programme, there are some on-going discussions on sub-contracting of facilities (for example, private facilities within the catchment area of an ‘official’ facility, which may be sub-contracted by the latter to carry out some services). In theory, the selection of sub-contracted facilities would be agreed on by the EUP.

In Zimbabwe, the accreditation system for facilities already existed prior to RBF and has not been changed by it. All public and mission facilities were accepted within RBF as long as they met the minimum managerial criteria, such as development of operational plans, having a functional HCC, having a bank account, and agreeing to remove user fees for mother and child health care [16].

Equally, in Uganda, prior systems for accreditation by the Ministry of Health and PNFP bodies, such as Uganda Catholic Medical Bureau, Uganda Protestant Medical Bureau, Uganda Muslim Medical Bureau, and Uganda Orthodox Medical Bureau, as well as annual licencing from the Medical Councils existed. RBF introduction additional quality criteria, which were used to incentivise and fund quality improvements, though focused on the PNFP sector in Uganda.

#### Establishing service agreements

Across the three settings, RBF introduced contracts at the facility level, which had previously not been in existence. For example, in DRC, under both RBF programmes, contracting is carried out by the EUPs. EUPs sign contracts with the districts and with health facilities, detailing indicators, tariffs, verification procedures and any other rules and tasks (e.g., preparation of business plans). The contracts are limited to the services covered by RBF and there is no evidence of this contractual approach having extended to other sectors (e.g. local government and the private sector) as yet. This is equally the case in Zimbabwe and Uganda.

In all contexts, these contracts are not enforceable and there is limited space for sanctions, or for negotiating contracts or excluding providers. So ‘contracting’ is rather weak. Providers sign the contract to get funds but without thinking too much about the details, according to key informants. It is also unclear how far the RBF contracts are permanently modifying behaviour – for example, the training on business plan development provides useful skills at facility level, but the plans are often not followed for lack of funds and it is unclear if providers would continue with these plans if not required to do so under RBF.

In these settings, formularies and standard treatment guidelines are provided by the Ministry of Health at central level, and RBF has not introduced any reforms. RBF programmes worked within the existing guidelines and standards: the list of indicators contracted and the quality checklist provided some reinforcement of national standards/guidelines by linking payment to criteria based on them.

#### Designing, implementing and modifying provider payment methods to encourage efficiency and service quality

In DRC, there has typically been underfunding or no funding at all for facilities or primary health care from the public budget. In addition, not all staff receives salaries: according to a public expenditure review, only 32% of the health workforce is included on payroll [36]. RBF has provided additional funds based on outputs, but focused on a limited set of indicators, and has not altered payment systems from government and other donors. In terms of its impact on quality and efficiency, there is little evidence. Under the FED project, while there was some improvement to equipment and infrastructure availability (structural quality) especially at hospital level, also thanks to the non-performance based component of the project, the impact on quality of services was negligible. This may be related to the fact that the project did not initially include quality indicators in the bonus calculations [19].

In Zimbabwe, the impact evaluation suggests a mixed picture in terms of gains in outputs and quality resulting from RBF [42]. The payments focus provider attention on maternal and child health services, however, many of the indicators are already high, in terms of coverage, so it is not clear how far the incentives are likely to be efficient (e.g. for antenatal care) [16]. RBF has certainly helped to reinforce national quality standards and has funded some quality improvements at facility level, such as filling gaps in drug supply. However, this is not necessarily the most efficient means to do that (local procurement costs being higher than national ones) [17].

In Uganda, there was some confusion at local level about payment methods and formulae, which were seen as complicated and unclear, as well as about the complex verification, involving expensive audit firms (who lacked medical expertise) [46]. The need to simplify, harmonise and reduce the cost of RBF programmes was raised. Key informants felt that implementers had developed payment systems alone, without consulting facility in-charges or PNFP representatives, and they did not understand how rates had been calculated or whether they reflected actual costs. In addition, key informants highlighted that it was hard for RBF to improve quality alone, given structural issues (procurement rules, wider funding issues, human resource allocation etc.).

#### Establish provider payment rates

In all three countries, RBF introduced specific payments for services, which was not the practice before (public services being financed through input-based budgets, generally inadequate). However, the way in which RBF indicators were set varied. For example, in DRC, under the FED programme, the choice of services and payment level was done by the CPP (provincial steering committee – therefore with some flexibility at provincial level), based on the budget available and preferences of the project funder, who approved the indicators, according to key informants. By contrast, under the PDSS programme, indicators included in the contracts are defined by the operational manual. Choices are said to be based on public health priorities and budget available. Also, the PDSS is focused on maternal and child health so most of the indicators cover those services. In theory, adaptations at provincial level are possible, but in practice they have not happened yet and all provinces follow what established in the operational manual.

In terms of tariffs, in both DRC's RBF programmes these represented an additional amount to (very low) existing funding [36]. Under the FED programme, tariffs were related to the real costs of service provision, while under the PDSS they are much lower, and aim to provide an incentive to lower user fees and increase coverage.

The RBF programmes are also facing challenges to ensure timely payments. For the FED programmes, there were delays in payment of up to three months [19]. The PDSS only started recently so information on payment delays is not yet available.

As with the PDSS programme in DRC, in Zimbabwe indicators were drawn up based on priorities and lagging indicators, focussed on RMNCH, as this was the focus for the funders. The payment is based on budgets and weightings across service levels, and provides marginal (not fully costed) payments to underfunded public facilities [16]. Indicators and rates are nationally established [20] and have been adjusted a few times, with the recent addition of some wider indicators for tuberculosis and HIV, though with very low payments which are unlikely to influence provider behaviour.

The initial assumption was that government would continue to provide base funding but that has not been realised, leaving primary facilities dependent on the RBF payments and hospitals under-funded [17]. This is causing concern, especially given the recent reductions in (and insecurity of) the RBF budgets, which have also caused delays in payments to facilities of three to six months.

In Uganda, the different schemes have had different indicators and payments, depending on the funder’s budget and area of interest. These could change and facilities be removed from the scheme based on unilateral decisions, often poorly communicated. Both government funds (for drugs and activities such as outreach) and RBF funds have been delayed by three or more months.

#### Allocating resources equitably across areas

The starting point in DRC was the absence of a resource allocation formula – or even resources – flowing to health facilities. RBF flows follow utilisation, not area-based needs assessment, but both RBF programmes incorporate a bonus element to compensate remote facilities. Under the PDSS, the bonus ranges between 0 and 40% based on the category of the facility, defined by its geographical accessibility (there are five categories from 1 for the most accessible ones to 5 for the remotest) [18].

Under both RBF programmes, extra funds are provided to facilities to cover the full costs of providing services to the very poor (*indigents*). Under the FED project, the mechanism was named *Fonds d’Equité* (equity funds) and was operated by external actors (NGOs, rather than EUPs), starting from 2008. The *Fonds d’Equité* only covered services at hospital level [45]. Under the PDSS, an increased tariff is paid to allow facilities to provide free services for the very poor, for a maximum of 5% of the population. However, the increase in tariff paid only affects one service at health centre level (curative visits) and three services at hospital level (days in hospital, major surgery, minor surgery). The very poor are pre-identified by the health committees, and lists are made available to the facilities [18].

Additionally, both RBF projects aimed to support facilities to lower user fees and/or introduce “flat fees” (which is also done by non RBF projects in DRC - [47]), thus cross-subsidising between more and less intensive patients. As there is no national regulation of user payment policies, fees are set by the facilities, in theory in collaboration with the health committee and the community. Both RBF projects aimed to better enforcing fees being defined in agreement with communities, publicly available and respected. They also aimed to reduce costs for the patients – at least for the services covered by RBF. Community verification surveys introduced under the RBF programmes include questions on fees paid by users. However, it is not clear what action is taken if fees are not respected. Overall, an internal evaluation of the FED found that fees were substantially lower thanks to the performance-based payments received by the facilities, but also the in-kind drug support. As a consequence, utilisation rates generally increased [19].

Zimbabwe also lacks a resource allocation formula, and budget allocations have followed historic patterns and planned activities [48]. While infrastructure is distributed relatively equitably in Zimbabwe, staffing is less so, especially for doctors [49]. The RBF programme provides some start-up funds and a remoteness bonus, but it is small and has failed to compensate for the small catchment populations which more remote facilities typically have [37]. Given the small payments per indicator, reaching the hard-to-reach populations is not well incentivised [17]. However, the RBF package is equitable in terms of its focus on essential services and primary level delivery, as well as its focus on enabling removal of user fees for MCH services [16].

RBF provided resources to support and reinforce a public policy of free services at primary level. However, some fees are still charged and the RBF evaluation did not find a difference in out of pocket payments between control and intervention areas [42].

Uganda has a capital development fund assigned to districts that are more disadvantaged, as well as a formula for allocating public funds that takes into consideration needs and populations [50]. However, resources such as staff are not equitably allocated and as with the other two countries, these major resources are not directly affected by RBF. RBF (BTC) provided some infrastructure investment for facilities that did not meet standards and early programmes such as NUHealth focused on post-conflict areas with higher needs. However, the majority of programmes, as highlighted, worked only with the private and PNFP sectors, and the selection of sites was often pragmatic, based on districts which were likely to be easier to work with. This has added to fragmentation across the system.

In relation to user fees, as with the other countries, reduction of fees was a precondition of most RBF schemes, especially those in the non-public sector. However, if RBF payments are low, it is difficult for facilities to reduce fees or stop charging [46]. Moreover, managing effective lowering of user fees will be more difficult if the pilots are extended to a larger area. In the case of the NuHealth scheme, although user fees were reduced in both areas, the majority of facilities in the intervention area continued to charge. Health care costs decreased in both Acholi and Lango [46].

#### Developing, managing and using information systems

In DRC, RBF operates a parallel information system to the national HMIS, which is considered to be weak. While HMIS data are entered by ECZ (health zone) staff based on facility reports, in the FED programme, the information system was based on verified data and managed at provincial level by the EUPs and the PDSS operates a centralised RBF Portal for the verified data. There are plans to ensure integration of HMIS/DHIS2 and OpenRBF, starting in end of 2018/early 2019, according to one key informant. While the implementation of the PDSS is too recent to be assessed, under the FED there were reported instances of mistakes and frauds by the facilities, which were introduced not only in the invoices but also in the facility’s registries and therefore also affected the HMIS system [19].

In terms of wider supervision, under the PDSS, zonal teams are also included in RBF and contracted by the EUPs to carry out a list of activities. These include monthly supervision to all facilities in their area [18]. In the FED programme, zonal teams had non-performance based funding from the central level only (not the RBF project) [19, 45].

In Zimbabwe, the RBF programme uses HMIS data, but having verified and corrected it, does not feed the data back into the HMIS (thus limiting its contribution to strengthening it) [16]. In general, in Zimbabwe, as in many settings, providers suffer from multiple data reporting requirements, registers, and surveys, and RBF has not eased the situation. Although it works from existing registers, by placing emphasis on exact recording – with sanctions for omissions, in the form of lost revenue – it adds to staff burdens and stress. The positive effect, however, is a greater focus on data quality. There is little evidence of false claims, so risk-based verification has been introduced. For procurement, public financial management procedures are very complex, making use of RBF funds time-consuming at facility level.

Zimbabwe had a well-developed and integrated supervision system prior to RBF, however, this was lacking resources after the economic crisis of the 2000s. RBF has provided funding to provincial and district teams which, though linked to RBF indicators, allows for wider supervision activities [16].

In Uganda, a similar issue of multiple data streams arises, although the HMIS is the main system for collecting data for health service delivery [32]. As in Zimbabwe, RBF programmes largely work within existing systems, and have both put additional demands on staff but also facilitated local improvements through providing funds for, for example, hiring more staff to help with data management. On the other hand, RBF auditors are not very skilled or trained, especially on clinical issues and data validators have also been on a learning curve.

District teams are also included in RBF in Uganda, and structural issues, like staffing capacity for supervision, are included in the performance indicators for districts in the NUHealth and SDS schemes (although it is difficult for the districts to act on them).

For the scaled-up model, there is still discussion of whether quarterly audits will be done by internal organisations or by an external one. As some perceived it, potential issues with fraud are overplayed in order to push for external agencies. It is likely that larger hospitals are at higher risk of fraud, compared to smaller units.

## Discussion

This article is an important addition to the literature, as it examines empirically – and for the first time, to our knowledge, in low income and fragile contexts - what the impact of RBF is on strategic purchasing in a health system as a whole. Strategic purchasing has been defined in many ways, but there is a consensus on some of its core features, and we use a detailed framework [8] to enable a structured examination. The three case studies present different contexts but many of the RBF design features are shared, leading to similarities of conclusion, as well as differences (summarised in Table 3).

Overall, the hypothesis that RBF would bring about widespread transformation of institutional relationships in the health financing and strategic purchasing architecture and be a catalyst for comprehensive health system reforms [5, 6] is not fully supported, although there are important gains in specific areas and subsets of services. At the government level, we find little change to the accountability of purchasers in these case studies, but RBF does mobilise additional resources to support entitlements. In relation to the population, RBF appears to bring in improvements in specifying and informing about entitlements for some services. However, the engagement and consultation with the population on their needs was found to be limited. In relation to providers, RBF did not impact in any major way on provider accreditation and selection, or on treatment guidelines (which has mixed implications – on the one hand, this may limit its power to raise standards, however set against that is the positive reading of that RBF was working in an integrated fashion within the existing health systems). However, it is important to note that RBF did introduce some critical changes in important areas of the purchasing systems. These include, a more contractual relationship for some providers and (at least partial) improvements in provider payment systems, moving away from (historical) budget or no funding at all towards output-oriented allocations, increasing the focus on data quality, increased financial management autonomy for primary providers and enforcing equitable strategies.

RBF remains an ‘add-on’ payment method [51] and cannot change all elements of strategic purchasing included in the analytic framework. It is also argued that RBF can have important effects in introducing change in strategic purchasing arrangements, for example in terms of output, quality and data focus, donor harmonisation and provider autonomy. However, these theoretical reflections hinge on the hypothesis that RBF is well designed and implemented, and well integrated in the health financing arrangements and existing systems [51]. In fact, we find that one of the reasons why RBF programmes may have had limited impact on overall strategic purchasing is that they have so far been viewed and implemented as stand-alone ‘financing mechanisms’ rather than as part of a mixed provider payment system, and have been run as pilot projects which are not integrated into the existing systems, including the health financing architecture [52]. This has led to a fragmentation and duplication of strategic purchasing actions under different programmes and schemes, which has diminished the potential for systemic change. Moreover, many schemes do not take up key elements and actions in relation to providers for payment systems, such as involving providers in their design, including risk adjustment and ensuring long term commitment [53]. Although, as some argue, RBF programmes can still represent a “first exposure” to strategic purchasing in terms of introducing the use of information in decision making and providing some financial and managerial autonomy to providers, our article highlights the outstanding challenges of integrating RBF into health systems to achieve reforms in overall strategic purchasing arrangements. These include aligning with other payment mechanisms, with wider public financial management and with verification systems [52], which is particularly challenging in fragile and donor-dependent contexts.

We also note that some of the key differences across case studies relate to the nature of the RBF programme, which may determine the different extent to which RBF integrates or reforms strategic purchasing arrangements and the health financing system more generally. For example, there are multiple pilots funded by different donors in Uganda and DRC, whereas in Zimbabwe, there is one national programme. In Uganda, the RBF programmes have hitherto focused on the PNFP sector, while in Zimbabwe and DRC, the main recipients are public sector providers (with a smaller PNFP component). Equally, the contextual differences are important to note and influence the degree of impact of RBF on strategic purchasing. Zimbabwe and Uganda have stronger government leadership in the sector, compared to DRC, and the under-funding of the sector is less extreme, both of which reduce the innovation space for RBF, while having other potential advantages, like increasing the likelihood of sustainability and integration, when buy-in is real [16]. Further, although all three have experienced recent conflicts and crises, the context is more stable in Uganda, in particular, but also to some extent in Zimbabwe, whereas DRC remains instable and conflict-affected, which tends to detract from longer term investments. Some of the programmes are young, though others, like the RBF programme in Zimbabwe, are operating on a national scale and now looking towards institutionalisation [12]. Clearly these findings represent a preliminary view of a changing landscape and more research would be needed in the future to document further developments.

The contextual differences also mean that recommendations are to be tailored to different settings. In particular, while institutionalisation and integration seem to be essential across all contexts to ensure that RBF plays a more significant role in reforming and strengthening strategic purchasing arrangements, the degree, speed and processes of such institutionalisation and integration should vary between settings, depending on the national leadership and stewardship capacity. In places where these are weak, such as the DRC among our cases, donors may need to play a more significant role in terms of supporting harmonisation processes (also through the creation of semi-autonomous purchasing entities). It is important that support for and development of strategic purchasing takes an overall system approach, with RBF as one tool amongst many to address systemic weaknesses.

### The experience of the EUPs in the DRC

Our analysis in DRC describes the experience of the EUPs, as semi-autonomous purchasing agencies, which represent an original model for the institutional design of RBF – somewhat similar to a recent proposal for the creation of Independent Service Authorities (ISAs) for service delivery in post-conflict, fragile states [54]. They also resemble the solution adopted in Cameroon where, after a series of RBF pilots which made use of an external agency for the implementation of RBF, the purchasing role was moved to a public organisation. A pre-existing body, the Regional Fund for Health Promotion was chosen and the purchasing functions transferred to it in 2014 [55]. These have the legal status of ‘public interest groups’ and are effectively regional dialogue structures, consisting of representatives of the communities, the MoH and public administration and donors. The inclusive composition of their membership aims to guarantee their accountability and also their independence from the government, making the RFHPs semi-autonomous bodies that can ensure the purchasing role is managed by a national agency, while maintaining a separation of function from the MoH [56].

In practice, EUPs are more closely linked to the needs of the implementation of the RBF programmes and have a narrow role in relation to purchasing. They do sometimes take up some of the roles related to strategic purchasing, such as verification, reporting, community feedback, and fund-holding (this last in the case of the FED EUPs), which are mostly functions which were not done at all previously. However, their decision-making power on other key elements related to strategic purchasing is very limited. Most activities remain in the hands of the government at national or provincial level, or are decided by the donor (such as regulation, definition of benefits package and level of tariffs, accreditation, even fund-holding and payment for the PDSS programme). The original vision of the EUPs becoming a joint, integrated pooling and purchasing agency at decentralised level for the entire health system (pooling revenues from different sources to purchase services) and also gaining financial and technical independence from external donors, remains unfulfilled so far. It will be relevant to conduct further research on their evolution under the ongoing RBF scheme.

#### Study limitations

This study drew on interviews whose number was more limited in some contexts than others, due to availability and accessibility of respondents. Equally, some of the documents which describe the process of policy development and roll out are confidential or not available, so while the researchers tried to access as broad a range of documents as possible, they could not be comprehensive. In particular, in DRC, interviews were carried out remotely and it was therefore difficult to reach many informants and in particular national actors. As a consequence, there is a clear predominance of international actors. Additionally, the PDSS programme is relatively recent and most of our documents and discussion with the informants are bound to refer only to its design and (very) early implementation. In Uganda, there have been several pilot schemes in the country that have not been well integrated into national health systems. The different schemes also had differences in design features, which makes studying the evolution of strategic purchasing arrangements difficult in the absence of a national RBF scheme. However, the diversity in schemes represents efforts to design RBF arrangements suitable for the Ugandan context and provided a rich perspective on different innovations in the purchasing function across the schemes.

Overall, during data collection care was taken to include all of the main stakeholders and participants in the programmes, not just present but over the programmes’ lifetime, and to cover all the documents available. As a result, we believe that our data collection and analysis has captured the key elements of interest for the selected RBF programmes in each setting, regardless of the number of interviews carried out and each case study is sufficiently relevant and rich to provide a meaningful comparative analysis.

## Conclusion

Using available secondary evidence and the insights from key informants who have been closely linked to the development of RBF programmes, we examine in this article preliminary evidence on how RBF programmes have affected strategic purchasing of health care in three low-income, crisis-affected countries – DRC, Zimbabwe and Uganda. We find that the RBF programmes do not seem to have brought about systematic transformation in the health financing and strategic purchasing architecture, and in some domains, particularly at government level and in relation to the population, have not altered arrangements towards more strategic purchasing. However, importantly, partial improvements are noted in some domains, such as creating more incentives for service delivery and quality for some services, while also bringing more focus to output-oriented allocations and data, provider autonomy, and enabling national policies to improve equity (such as user fee removal or reduction) to be at least partially implemented. More generally, RBF has been a source of much-needed revenue at primary care level in under-funded health systems. The evidence to date suggests that expectations of RBF should be nuanced as RBF remains an add-on component of payment systems, while focusing on expanding areas of potential gain and ensuring better integration and institutionalisation, which some of the countries described here are starting to work towards.

## Annex 1

### The EUP model for strategic purchasing in DRC

The establishment of the *Etablissements d’utilité publique* (public service agencies - EUPs) in relation to RBF implementation in DRC is an innovation in terms of institutional arrangements for the strategic purchasing compared to the more commonly adopted external agencies (international NGOs or implementing agencies), or in a few cases Ministry of Health (MoH) structures. In this box, we provide further details on their creation, their institutional arrangements and functioning mechanisms.

Four EUPs were created in 2008, the provinces where the FED programme operated.^5^ They were composed of a Director, an administrator and a pool of verifiers. Their main tasks were contracting facilities (although it is recognized that negotiation space was very limited), carrying out verification procedures (checking quantity of services provided, and contracting and managing community-based organisations for community verification), calculating payments owed to each facility, and paying providers. Based on the latter task, EUPs also played a fiduciary role at provincial level in holding EU funds and allocating them to facilities. In terms of institutional organisation, the EUPs were governed by a Board of Directors (*Conseil d’Administration*, CA) composed of representatives at provincial level of the MoH (DPS), the Ministry of Finance, the General Secretary of the government, donors, NGOs, civil society (for example, Catholic Church and/or other organisations), as well as the director of the EUP. The CA had a role of strategic orientation and oversight of the EUP. Additionally, a Provincial Steering Committee (*Comité Provinciale de Pilotage,* CPP) was in place composed of the Director of the EUP, the DPS, as well as technical assistants and NGOs involved in the implementation of the FED project,^6^ with a more technical role in relation to the implementation of the RBF programme and of the strategic purchasing choices (for example, including inclusion/exclusion of facilities, RBF’s list of indicators and level of payment to providers) [45].

The PDSS programme opted to make use of the EUP model as a strategic purchasing agency for RBF. The reasons for this choice are again related to the potential of the model to ensure the participation of different representatives, including of government and MoH, but also of donors, NGOs and civil society, while at the same time guaranteeing management autonomy, as well as to improve the ownership and sustainability of the purchasing agency [18]. The EUPs’ role as strategic purchasing agency is emphasized in the PDSS Operational Manual. Indeed, under the PDSS, the RBF approach is often referred to as ‘strategic purchasing’, with the two terms almost considered synonymous throughout the Operational Manual. This points to the role assigned to RBF in relation to the purchasing function, as an (almost automatic) enhancer of strategic purchasing within a system.

New EUPs have been created in the provinces where the PDSS operates and have a similar organisation to the EUPs created under the FED programme, with a small team assigned to each EUP which includes the verifiers, and a CA composed of representatives of public sector, civil society, donors and NGOs overseeing its work. The CPP is now merged with the Technical Working Group on Health Financing and Universal Health Coverage (*GTT Financement*), which is a technical body composed of the Provincial Health Authorities (DPS), donors and NGOs already operating at provincial level. The *GTT Financement* has a broader health system and UHC focus not only limited to the management of the RBF programme, but also in charge of RBF activities, such as reviewing the payments to be made each quarter and discussing the results and challenges [18]. While the EUPs created by the PDSS are in charge of contracting facilities and carrying out verification, compared to the EUPs under the FED programme they do not have the fund-holding and payment role (and therefore the original fiduciary role), which has now been moved to central level. Some of the key informants reasoned that this may be because the new EUPs do not have any technical assistants embedded in their structure (as the FED project EUP did) and therefore it may be riskier for the donor to allow them to manage funds directly. Others also stressed that the IT system is now centralized through a RBF Portal, which in turn allows for a centralized payment system. In addition, a second layer of verification (‘external verification’) has been added by contracting an international agency to carry out counter-verification of a random sample of facilities every 3 months.

## Abbreviations

BTC: Belgian Technical Cooperation
CA: Board of Directors (*Conseil d’Administration*)
CPP: Provincial Steering Committee (*Comité Provinciale de Pilotage*)
DFID: UK Department for International Development
DPS: Provincial Health Divisions (*Divisions Provinciales de Santé*)
DRC: Democratic Republic of Congo
ECZ: Zonal Health Teams (*Equipes Cadres de Zone)*
EU: European Union
EUPs: E*tablissements d’utilité publique* (public service agencies)
FCAS: Fragile and conflict-affected states
FED: *Fonds européen de développement* (European Development Fund, EU RBF programme in DRC)
GAVI: Global Alliance on Vaccines Initiative
*GTT Financement*: Technical Working Group on Health Financing and Universal Health Coverage (*Groupe Technique de Travail Financement and Couverture Sanitaire Universelle*)
HCC: Health Centre Committee
HDF: Health Development Fund
HMIS: Health management information system
ISA: Independent Service Authority
KI: Key informants
MCH: Maternal and child health
MoH(CC): Ministry of Health (and Child Care)
NGO: non-governmental organisation
PBF: Performance based financing
PCA: ‘complementary package of activities’ for hospitals (DRC)
PDSS: *Programme de Développement de Services de Santé* (World Bank RBF programme in DRC)
PIU: Project Implementation Unit
PMA: ‘minimum package of activities’ for health centres (DRC)
PNFP: Private not-for-profit
RBF: Results-based financing
RBM: Results-based management
RHC: Rural health centre
RMNCH: Reproductive Maternal Neonatal and Child Health
SDS: Strengthening Decentralisation for Sustainability (health and RBF programme in Uganda)
SIDA: Swedish International Development Agency
SMGL: Saving Mothers, Giving Lives (RBF programme in Uganda)
UNDP: United Nations Development Programme
UNFPA: United National Fund for Population Activities
UNICEF: UN Children’s Fund
USAID: United States’ Agency for International Development

## Footnotes

1 Health Zones in North and South Kivu are covered by RBF funded by the World Bank, but under different funding (not PDSS) and slightly different model.

2 As a key informant noted, the EUPs created at provincial level for the purchasing of health services were called “*Fonds d’Achat de Services de Santé*” (Health Service Purchasing Funds) but this name did not stuck and instead the term defining their legal status, rather than their role, remained in use.

3 According to the MOH website, RBF is one of the strategic options under the Institutional Capacity Building (ICB) Project Phase 2. RBF, which was only with PNFP facilities in Phase one of the ICB project will, under phase 2, be expanded to public health facilities. Implementation will feed national policies for future social health insurance system development in the country. Accessed from: http://health.go.ug/projects/institutional-capacity-building-phase-ii

4
www.fbp-rdc.org
